# The effect of teaching integrated course of physical examination and radiological anatomy in practical limb anatomy on medical students’ learning outcomes

**DOI:** 10.1186/s12909-021-02898-z

**Published:** 2021-08-30

**Authors:** Hossain Sadeqi, Ali Valiani, Maryam Avizhgan, Seyed Abbas Ebrahimi, Amirreza Manteghinejad, Pantea Miralai, Athar Omid

**Affiliations:** 1 Department of Anatomical Sciences, School of Medicine, Isfahan university of medical sciences, Isfahan, Iran; 2 Medical Education Research Center, Isfahan University of Medical Sciences, Isfahan, Iran; 3 School of Medicine, Isfahan University of Medical Sciences, Isfahan, Iran; 4 Student Research Committee, School of Medicine, Isfahan University of Medical Sciences, Isfahan, Iran; 5 Department of Medical Education, Medical Education Research Center, Isfahan University of Medical Sciences, Isfahan, Iran

**Keywords:** Anatomy, Physical examination, Radiological anatomy, Clinical Integration

## Abstract

**Background:**

In the last few decades, the need to change the curriculum of basic medical science has been further emphasized. The purpose of this study was to evaluate the effects of teaching integrated course of physical examination and radiological anatomy in practical limb anatomy on medical students’ learning outcomes.

**Methods:**

This was an experimental study. Medical students (of the 4th semester of medical education) were divided into intervention and control groups. Related topics of physical examination and radiological anatomy were added to the practical limb anatomy courses of the intervention group. Practical knowledge of anatomy, clinical applications of anatomical knowledge, students ‘satisfaction, and students’ attitude toward the anatomy course were assessed at the end of the study. Knowledge retention was assessed three months after the semester.

**Results:**

The intervention group scored significantly higher mean scores in practical knowledge of anatomy test, clinical applications of anatomical knowledge test and knowledge retention test (P-value < 0.05). In evaluating students’ satisfaction with the course, the intervention group was satisfied with the course and teacher performance and had appropriate attitude (Mean˃4, Max score = 5) towards the application of anatomy in medicine.

**Conclusions:**

The findings of this study showed that teaching practical anatomy with a clinical integrated approach can improve the practical knowledge of anatomy, knowledge retention, and clinical applications of anatomical knowledge. In addition, an integrated approach was associated with greater student satisfaction and it makes students have appropriate attitude towards the application of anatomy in medicine.

## Background

Extensive reforms in the education of general medicine have a long history. Most of these are because of two facts; the first one is the different learning methods of the new generation. The other one is the major advances that have been made in medical imaging. In this situation, the use of slides and lectures cannot meet the needs of students (1). Alongside every other course, teaching of anatomy was also affected. In 1993, The General Medical Council of the United Kingdom’s executed fundamental reforms in the basic medicine teaching. The aim and the result of these reforms was to increase the time devoted to clinical and practical teaching and to reduce the teaching time of basic sciences (2–4). The physicians graduated from the new system had lower knowledge of basic medical sciences but were more skilled in clinical practice (5–7). Following these reforms, the use of cadaver was declined because it was time consuming, costly, and it was shown to have had little effect on medical students learning of anatomy (8).

Further studies have shown that if students are taught a large amount of basic science content in a non-practical way, it will lead to superficial learning and rapid forgetfulness. Also, lack of attention to the needs and interests of learners will reduce their motivation for learning basic science courses, including anatomy (8, 9). Therefore, researchers have recommended that in learning anatomy, students should integrate basic and clinical sciences so that they can use them effectively on the patient’s bedside (9).

However, these reforms in basic science education have raised the concerns that this trend may lead to a decline in teaching of the basic sciences and its gradual removal from the medical science curriculum (10). In the response to this issue a new perspective emerged that the basic and clinical sciences are not separate fields, basic sciences should be the basis for learning clinical skills. Therefore, it is important to emphasize content that enhances clinical skills (11, 12). In addition, studies have shown that the most appropriate way to teach anatomy is using combination of several strategies and teaching methods. These include case based discussion, course integration, using medical imaging, etc(8). Accordingly, various studies have examined the effect of these methods and strategies and the results of these studies have been often contradictory (13–15). The purpose of this study was to investigate the effect of teaching integrated course of physical examination and radiological anatomy in practical limb anatomy on medical students’ learning outcomes.

## Methods

This study was an experimental, case-control study that conducted in the first semester of 2019. This research approved in Isfahan University of Medical Sciences and all methods were performed in accordance with the relevant guidelines and regulation**s.** The research environment was Isfahan University of Medical Sciences, and all the students who enrolled in the course “Practical Anatomy of Upper and Lower Extremities” were the study population. The practical anatomy course is one of the essential units of basic medical sciences and all medical students are required to pass this course. This course is offered in the 4th semester of medicine.

After approval by the ethics committee and deputy of education, all the students who enrolled this course in the first semester of 2019 were randomized by a random allocation software into four groups of 14 to 16 students. Two groups were placed into the intervention group, and two groups were placed into the control group. .Throughout the semester, in the traditional method, osteology was first taught using bone and modeling.

The nerves, arteries, and muscles on the model were taught to the students of the course.Finally, radiology images and visible elements were briefly taught, for example, bones were shown on foot graphs. .

In the integrated method with the clinic, all these cases were taught in half of the usual time, and in the second half of each class, clinical content, physical examinations and radiology anatomy were taught in the same topics of the first half of the class.

**Fig. 1:**
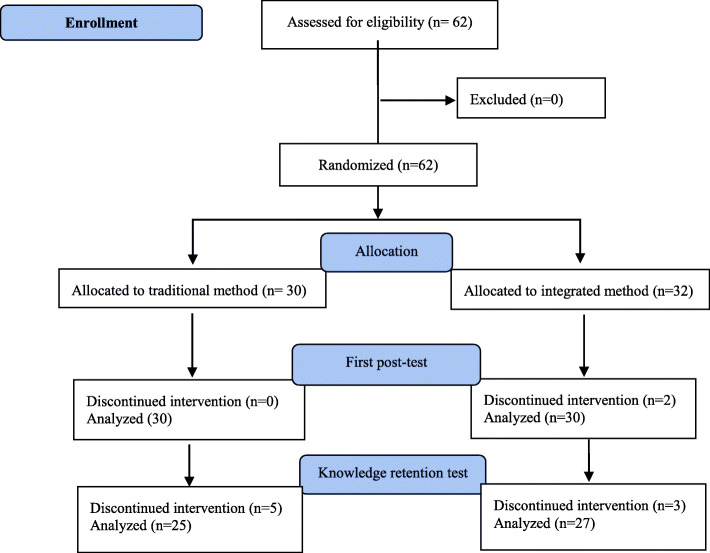
The flow diagram of the study

Inclusion criteria were choosing practical limb anatomy course in the first semester of 2019 academic year. The exclusion criteria were more than two session absences and failure to participate in the final test. Flow of the research shows in the diagram (Fig. 1).

Throughout the semester, this course had 13 sessions; each was 2 h. For the intervention group, each session divided into two parts. In the first part, students received content similar to the control group. This content included osteology, learning on cadavers, and some points about surface anatomy and radiologic anatomy. This was based on Iran’s ministry of health and medical education curriculum. In the second part, the teacher integrated clinical points based on the first part, radiologic anatomy in more depth, and teaches students physical examination. The control group received only the content of the first part within two hours. The first part was taught by a faculty member who was the same between the intervention and control groups. The second part was taught by a medical intern who is the same in the 2 intervention groups.

Six quizzes were conducted during the course to assess students’ practical knowledge of anatomy. Each quiz had three stations: osteology station, mannequin station, and cadaver station. The order of the stations was the same for all the students. All the quizzes were oral. For example, the student was asked to show the radial nerve canal in the humerus bone or at a cadaver station, a particular nerve or muscle was shown to the student and the name of that nerve or muscle was asked. This test was designed by an anatomy teacher who was not part of the teaching group and the research team and did not know about the intervention and control groups. To prevent bias, the assessors were selected from postgraduate students in anatomical sciences who were not in the teaching team and were not familiar with the intervention and control groups.

The test for assessing clinical applications of anatomical knowledge was held in the eighth (end of upper limb anatomy course) and fourteenth (End of lower limb anatomy course) weeks for both control and intervention groups. A member of the research team designed the questions of this section. The questions were based on the second part of each session of the intervention group and were short answer, modify essay and extended matching. A case was designed in the stem of the questions and in each case, the student was asked to write the name of the injury (dislocation, fracture, ligament injury, etc.), complications and anatomical mechanism of the injury. In another part of the test, a graph of a fracture or dislocation was shown and students were asked to identify the type of injury and write the complication of the injury by explaining the cause. For example, a picture of a humeral shaft fractures was shown, and in addition to diagnosing the fracture, the student was expected to point to a wrist drop due to radial nerve damage. The exam was scored by giving a code to the worksheets and the intervention and control subjects remained unfamiliar to the evaluators and the final grades of the intervention and control group were compared together. Regarding the anatomy application test in the clinic, the questions were designed in such a way that the control group could answer them by analysis if they learned the concepts of anatomy in depth.

The purpose of designing this test was to prove that not only have students learned practical material, but that they have learned nothing less than learning pure anatomy, even better and more durable.

For assessing the knowledge retention three months after the semester, another exam was done. The questions were the same six practical anatomy quizzes. The assessment was done without any prior announcement to students. Participating in this exam was voluntary. The questions and the order of rotation at the stations were the same for all students. The assessors were not familiar with the intervention and control groups. The questions were selected by one of the anatomy professors outside the research team. The mean responses of the two groups to the questions in the intervention and control groups were compared. All scores are calculated from 20.

For the attitude assessment of the case group toward the course, a valid and reliable questionnaire was used. Adibi et all designed the questionnaire (16). The questionnaire has 11 questions on a Likert scale (1 to 5). This questionnaire had the Cronbach’s alpha of 0.94. For the satisfaction assessment of the case group toward the course and teachers’ performance, a questionnaire was designed by the research team. The questionnaire has 13 questions based on the Likert scale. The questionnaire had Cronbach’s alpha of 0.79.

All the data were analyzed by an SPSS version 16 software. For the comparison between the case and control group paired T-test was used.

## Results

In this study, which was conducted in 2019 at Isfahan University of Medical Sciences,62 people entered the study, of which 29 (46.8 %) were women and the rest were men. There were 32 people (15 women) in the intervention group and 30 people (14 women) in the control group. During the study, two members of the intervention group were excluded from the posttest analysis due to the absence of more than 2 sessions. Following the study five members of control group and 3 members of intervention group were excluded from the knowledge retention test analysis due to not participating in test.

The mean scores for the clinical applications of anatomical knowledge test and practical knowledge of anatomy test in both intervention and control groups are shown in Table 1. In both tests, the mean for the intervention group was higher and the difference in the scores was significant (*P*-value < 0.05).

**Table 1:** Mean test scores of control group and intervention group and significance level

	Groups	Mean (max 20)	Standard deviation	*P*-value
Average of academic achievement in prior semesters	Intervention group (*N* = 30)	15.47	1.40	0.81
	Control group (*N* = 30)	15.38	1.56	
Clinical applications of anatomical knowledge	Intervention group (*N* = 30)	16.56	2.04	0.00
	Control group (*N* = 30)	4.88	2.08	
Practical knowledge of anatomy	Intervention group (*N* = 30)	17.79	1.37	0.01
	Control group (*N* = 30)	16.50	2.36	
Knowledge retention 3 months later	Intervention group (*N* = 27)	11.55	5.62	0.00
	Control group (*N* = 24)	2.50	2.13	

Average of academic achievement in prior semesters as a disruptor variable was also considered, which no statistically significant difference was observed between the two groups (*p* = 0.81). Knowledge retention was assessed in both groups after 3 months that had significant difference (*P* = 0.00). (Table 1)

The student’s satisfaction with the practical anatomy course and teacher’s effectiveness are shown in Table 2. The intervention group was satisfied with the educational content (4.37 ± 0.83), the attractiveness of the topics (4.59 ± 0.91), the use of different learning activities (3.87 ± 1.21), proper teachers’ expression (4.71 ± 0.72), clinical applicability of the subjects (4.93 ± 0.24) and quality of teamwork (4.71 ± 0.58).

**Table 2:** Mean and SD of students’ satisfaction with the practical anatomy course and teacher’s performance in intervention group

Item	Intervention group
Quality of teamwork	$4.71\pm 0.58$
Teachers’ knowledge of the subjects	$4.62\pm 0.75$
Proper teachers’ expression	$4.71\pm 0.72$
Clinical applicability of the subjects	$4.93\pm 0.24$
Classroom management	$4.78\pm 0.42$
Adequacy of physical space	$3.71\pm 0.58$
Appropriateness of content	$4.37\pm 0.83$
Appropriate sequence and order of content presentation	$3.65\pm 0.82$
Adequate session’s time	$3.53\pm 1.21$
The attractiveness of the topics	$4.59\pm 0.91$
Variety of learning activities	$3.87\pm 1.21$
Suitability of light, ventilation and	$3.62\pm 0.94$
Appropriateness of audio-visual facilities	$3.28\pm 1.11$

The mean and standard deviation for clinical attitude in intervention group is shown in Table 3. As it is shown, the average score for clinical attitude is higher than 4 and the students had appropriate clinical attitude when integrating physical examination course with practical anatomy.

**Table 3:** Mean and SD for clinical attitude in intervention group

	Clinical attitude score(max5)
I prefer to learn clinical subjects alongside the basic subjects in basic science course	$4.68\pm 0.69$
Using clinical and practical lessons in teaching anatomy improved my understanding from basic anatomy lessons	$4.75\pm 0.67$
Using clinical and practical lessons in teaching anatomy improved my ability to learn anatomy	$4.93\pm 0.24$
Using clinical and practical lessons in teaching anatomy increased my motivation to learn anatomy	$4.59\pm 0.79$
Teaching clinical and practical lessons in teaching basic sciences is necessary for my education	$4.78\pm 0.75$
Learning clinical and practical applications of anatomy in basic science course is a valuable tool in my future clinical practice	$4.90\pm 0.39$
New teaching methods helped me to pay more attention to clinical subjects	$4.53\pm 1.13$
I think the new teaching method is necessary for all the basic science courses	$4.68\pm 0.85$
It is easier to remember the anatomy contents with the integrated teaching method than the classic method	$4.65\pm 0.74$
In future, I will use the integrated method when studying basic sciences even if there are no integrated courses in our education	$4.40\pm 0.94$

## Discussion

This study was conducted to evaluate the effect of teaching integrated course of physical examination and radiological anatomy in practical limb anatomy. The practical knowledge of anatomy, knowledge retention, student’s satisfaction and student’s clinical attitude were calculated. The intervention group scores for practical knowledge of anatomy test, clinical applications of anatomical knowledge test and knowledge retention test were higher significantly. And they also were satisfied from most of the items in the satisfaction questionnaire. The average score for clinical attitude was higher than 4 in the intervention group which indicates positive attitude of the intervention group towards anatomy lessons.

Supporting our data, Hersh et al. have shown that if the basic science theory are integrated with clinical and practical content, the student will be more prepared to enter clinical environments and communicate with patients and they are more satisfied. In addition, the knowledge retention was higher compared to traditional methods. (17, 18).

Medical education had always been consisted of two parts, basic sciences and clinical education. Gradually, the importance of basic sciences in helping to solve clinical problems has received more attention. Numerous studies have shown that anatomical knowledge is necessary for a physician’s daily activities, including physical examination, interpretation of para-clinical tests, hypothesis evaluation and treatment procedures (19, 20). While in the traditional method, teaching practical basic sciences was more about the basic subjects and less about its clinical applications. It is expected during clinical education, the basic sciences with clinical sciences will be integrated (21).

The cohort study of Atticah et al. in 2019 on 183 medical students from the first year to fifth year have shown that most of the students in both basic and clinical courses were aware of the importance of anatomy knowledge in clinical practice. They believed that the anatomy knowledge is essential for physical examination, invasive procedures and surgery. In their study the main factors affecting students learning were insufficient evaluation, non-integrated courses and inappropriate teaching tools and methods. Students’ opinions in both clinical and basic courses suggested that integrated teaching of clinical lessons along with basic science was much more effective (22). The results of this study and other studies on the matter suggested that teaching basic sciences with a clinical base were more understandable for students. The students could apply their knowledge better and more easily in a clinical setting (22, 23). Another study on American students in 2017 found that teaching anatomy in a clinical simulation environment was very effective and can lead to excellent short-term knowledge retention. (24).

Other studies on Iranian students also support our data on student’s clinical attitude after teaching integrating course. These studies showed that the good expression of the teacher, using of various methods and teaching aids. In addition to teaching basic and clinical sciences simultaneously will lead to better learning and more satisfaction and improves student’s motivation and attitude. They also showed, if the content of teaching courses is more aligned to medical student’s professional requirements, the students will be more interested in learning and participate better in class (16, 25, 26).

One of our differences with other studies on this matter was that the knowledge retention in addition to clinical applications of anatomical knowledge and practical knowledge of anatomy were assessed. It is important to note that the main objective in this study was not to teach clinical content to students, but to use clinical content as a tool for better and more knowledge retention.

However, it should be noted that teaching integrated course of physical examination was implemented in excess of what students expected from practical limb anatomy course. Their practical anatomy score was recorded as their final score. But it was not possible to assess how much this concern about the final score caused the intervention group to make more efforts to get a better grade. On the other hand, the facilities available in the anatomy hall did not meet the teaching needs of clinical subjects such as physical examination, superficial and radiological anatomy. Perhaps teaching clinical content in a more appropriate environment for learning physical examination such as a clinical skills learning center (skill lab) will help students learn better. The control group and the intervention group were related in other classes and there was a possibility of interference. In this study, it was tried that none of the members of the control group was present in the anatomy hall while teaching clinical content to the intervention group.

## Conclusions

Teaching anatomy as one of the important basic sciences for clinical practice must improve over time and requires the use of new teaching methods. The findings of this study showed that integrating physical examination and radiological with practical anatomy not only does not reduce the practical knowledge of anatomy but also improves the clinical applications of anatomical knowledge, knowledge retention. In addition, an integrated approach was associated with great student satisfaction and it makes students have appropriate attitude towards the application of anatomy in medicine. Therefore, it is recommended that the teaching of limb anatomy in the basic sciences be integrated with the teaching of physical examination and radiological anatomy. It is also suggested that in another cohort study, the effect of integrating teaching physical examination with practical anatomy on students’ clinical skills in the clinical setting should be investigated.

## Data Availability

The datasets used and/or analyzed during the current study available from the corresponding author on reasonable request. Authors can confirm that all relevant data are included in the article and/or its supplementary information files.
